# Temporal Vulnerability of the Blood-Brain Interface in Stroke: Molecular Mechanisms of Circadian Dynamics, Inflammation, and Aging

**DOI:** 10.3390/ijms27135729

**Published:** 2026-06-25

**Authors:** Sarah Asif, Jennifer W. Mitchell, Martha U. Gillette

**Affiliations:** 1 Neuroscience Program, University of Illinois Urbana-Champaign, Urbana, IL 61820, USA; sasif3@illinois.edu (S.A.); mitchll3@illinois.edu (J.W.M.); 2 Beckman Institute for Advanced Science & Technology, University of Illinois Urbana-Champaign, Urbana, IL 61801, USA; 3 Bi[o]hub, Chicago, IL 60642, USA; 4 Department of Cell & Developmental Biology, University of Illinois Urbana-Champaign, Urbana, IL 61801, USA; 5 Department of Molecular & Integrative Physiology, University of Illinois Urbana-Champaign, Urbana, IL 61801, USA

**Keywords:** stroke, circadian rhythms, blood–brain interface, inflammation, aging

## Abstract

Stroke remains one of the leading causes of long-term disability and death worldwide. Growing evidence suggests that both stroke onset and severity exhibit strong circadian patterns. This blood–brain interface (BBI), which regulates bidirectional communication between the peripheral circulation and the central nervous system, plays a critical role in cerebrovascular injury. Aging further exacerbates these processes by dampening the molecular clock function and increasing inflammatory activation. In this review, we examine the circadian regulation of the BBI, aging, and its implications in stroke vulnerability. Understanding how circadian biology modulates neurovascular function may reveal novel therapeutic targets and time-of-day-dependent approaches for stroke prevention and treatment.

## 1. Introduction

Stroke is one of the leading causes of death and long-term disability worldwide [1]. The World Stroke Organization estimates that one in four adults over the age of twenty-five will experience a stroke in their lifetime. Aging remains the most significant non-modifiable risk factor, with stroke risk increasing sharply over the age of sixty-five [2]. Globally, the economic burden, including acute medical care, long-term rehabilitation, and productivity loss, exceeds USD 890 billion annually [2,3]. Given its staggering global impact and multifactorial etiology, stroke represents a paradigmatic disease in which vascular integrity, inflammatory response, and systemic circadian rhythms converge. Understanding interactions among these multiple regulators is poorly understood.

Historically, traditional frameworks have largely treated stroke as a static vascular event, without consideration of temporal context. However, accumulating evidence reveals that its occurrence is largely shaped by daily rhythms [4,5]. Many epidemiological studies have demonstrated robust circadian patterns in stroke onset, and newer studies have highlighted that the time of stroke incidence shapes stroke severity [6,7]. These observations suggest that endogenous circadian rhythms may create dynamic windows of heightened cerebrovascular vulnerability. These rhythms not only shape when strokes occur, but also their severity once initiated.

Understanding these time-dependent patterns requires re-examining the neurovascular system. The blood–brain interface (BBI), the brain’s first line of defense, is emerging as a critical structure linking circadian regulation to cerebrovascular injury. The BBI is increasingly recognized as a highly dynamic and rhythmic interface rather than a static wall [8,9]. Not only does the BBI change over the circadian cycle, but aging can further shape the BBI, compromising barrier structure and function. These age-related changes may diminish the rhythmicity and resilience of the BBI, potentially widening windows of vulnerability and contributing to worse stroke outcomes in older individuals [10,11]. Throughout this review, we use “blood–brain interface” (BBI) to reflect its dynamic, multicellular, and regulatory nature. The term “blood–brain barrier” (BBB) is retained where we reference historical literature and prior studies that used that terminology.

In this review, we synthesize evidence on the current understanding of molecular inflammatory mechanisms by which circadian timing and aging regulate BBI function. By framing stroke as a circadian-modulated disorder, we highlight the emerging concept of temporal vulnerability that can be harnessed as an opportunity for chronotherapy [12,13].

## 2. Circadian Modulation of Stroke Risk and Severity

### 2.1. Circadian Rhythms

**Figure 1:**
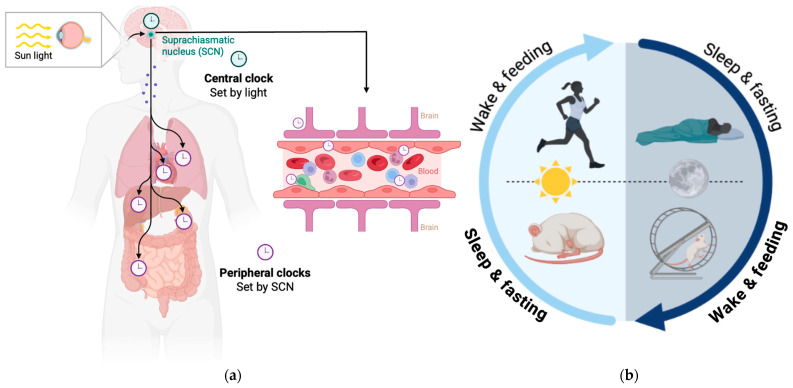
Schematic of circadian organization and behavioral rhythms. (**a**) The central circadian clock located in the suprachiasmatic nucleus (SCN) of the hypothalamus is entrained by environmental light cues. Photic input synchronizes molecular clock oscillations within the SCN, which coordinates peripheral clocks across multiple organs. Coordination occurs via neural, hormonal, and autonomic pathways. Critically for the blood–brain interface, the vascular endothelium harbors its own peripheral clock that is synchronized by the SCN and governs time-of-day-dependent regulation of barrier properties and immune cell trafficking. The inset depicts a cross-section of a cerebral vessel, showing endothelial clock expression (clock icons) and the circadian regulation of leukocyte presence within the blood compartment, reflecting rhythmic immune cell trafficking at the BBI; (**b**) Behavioral and physiological rhythms across the near-24 h light-dark cycle in rodents and humans. Rodents are nocturnal, with the active phase (wake/feeding) occurring during the dark period, whereas humans are diurnal, with peak activity during the light period. These opposing activity patterns reflect species-specific circadian alignment while preserving conserved molecular clock mechanisms. Created with Biorender.com.

Circadian rhythms are evolutionarily conserved, near 24 h oscillations that govern physiological, metabolic, and behavioral processes across almost all organisms. In mammals, these rhythms are coordinated by the central master clock within the suprachiasmatic nucleus (SCN) located in the anterior hypothalamus [14]. Light information is received by melanopsin-bearing retinal ganglion cells, entraining the SCN, and enabling environmental day-night cycles to align internal biological time. Through this organization, the SCN can synchronize peripheral clocks within cells and tissues via neural, endocrine, and autonomic outputs (Figure 1) [15,16]. Best recognized for organizing the timing of the sleep–wake cycle, it also exerts its effects on hormonal secretion, heart rate, the blood–brain interface, and inflammatory processes [16,17]. A key hormonal mediator of this process is melatonin, synthesized and secreted by the pineal gland under SCN control. Melatonin production rises during the dark phase and is suppressed by light, serving as a circulating signal of darkness that entrains peripheral clocks throughout the body and reinforces the alignment between central and peripheral circadian timing [18,19]. Recent studies have uncovered links between circadian rhythms and the BBI, discussed in Section 4 of this review.

**Figure 2:**
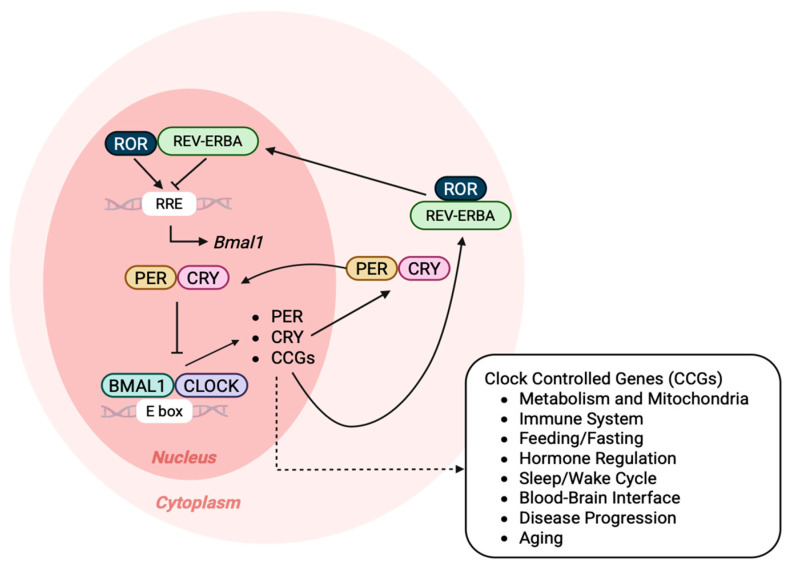
The transcription-translation feedback loop (TTFL) of the molecular clock: BMAL1 and CLOCK heterodimerize and bind to E-box response elements, promoting transcription of Period (PER), Cryptochrome (CRY), and additional clock-controlled genes (CCGs). PER and CRY accumulate in the cytoplasm, dimerize, and translocate back into the nucleus, where they inhibit the BMAL1-CLOCK-mediated transcription. Auxiliary regulatory loops involving ROR and REV-ERB nuclear receptors modulate BMAL1 transcription via ROR-binding elements (RRE). Clock-controlled genes regulate diverse physiological processes, including metabolism, immune function, feeding/fasting, hormonal regulation, sleep/wake cycle, BBI integrity, aging, and disease progression. Created with Biorender.com.

On the molecular level, circadian rhythms are driven by a cell-based molecular clock that emerges from dedicated clock genes [20,21,22]. This rhythmic cycle of transcription-translation gives rise to gene networks exhibiting oscillations with near-24 h periods. The core molecular clock consists of a set of core clock genes forming transcription-translation feedback loops (TTFLs). Specifically, the transcription-translation of the core clock genes BMAL1 (brain and muscle Arnt-like protein 1) and CLOCK (circadian locomotor output cycles kaput) leads to the heterodimerization of the BMAL1-CLOCK complex in the cytoplasm, which initiates nuclear translocation and binding to the canonical Enhancer Box (E-Box) gene promoters on the DNA [14,15,22]. BMAL1 and CLOCK induce transcription of their negative regulators, PER (PERIOD) and CRY (CRYPTOCHROME). The resultant PER-CRY heterodimer translocates to the nucleus, where it interacts with the BMAL1–CLOCK heterodimer to inhibit transcription (Figure 2). The cycle recommences as the transcription of PER-CRY diminishes due to reduced BMAL1-CLOCK levels [14,15,22].

### 2.2. Circadian Patterns of Stroke Onset

The concept that the occurrence of cerebrovascular events in the human population exhibits a circadian pattern was first introduced in the late 1970s, when Marshall (1977) [23] published a report describing diurnal variation in stroke incidence. In this early analysis, Marshall noted a surprising predominance of infarctions occurring between midnight and 6 a.m., suggesting that biological rhythms may influence cerebrovascular vulnerability [23]. Although these results contradicted later findings, they were pivotal in framing stroke as a temporally patterned disorder rather than a random event.

During the 1980s, systematic hospital-based reports began to clarify the temporal distribution of stroke onset. Tsementzis et al. (1985) studied over 550 patients and demonstrated a clear peak in stroke occurrence between 10:00 a.m. and noon, particularly for thromboembolic infarctions, intracerebral hemorrhage, and subarachnoid hemorrhage [24]. This was among the first comprehensive datasets linking morning hours to increased stroke onset. This coincides with known circadian surges in arterial pressure, heart rate, and platelet aggregation [24]. Soon after, Marler et al. (1989) provided robust evidence for a morning increase in ischemic stroke onset in a cohort of over 1000 patients, confirming that cerebrovascular events cluster in the early part of the day, specifically 10:00 a.m. to noon [25]. Together, these studies established the notion that circadian physiology, rather than external triggers alone, modulates cerebrovascular risk.

In the early 1990s, prospective cohort studies deepened the temporal analysis of ischemic stroke. Marsh et al. (1990) reported that nearly 60% of ischemic strokes occurred between 6:00 a.m. and noon, with roughly a quarter presenting within the first hour after waking [26]. These data aligned stroke onset with the “morning surge” in sympathetic tone and blood pressure. Kelly-Hayes and colleagues (1995) extended these findings in the community-based Framingham Study, which revealed a similar morning predominance (8:00–12:00 p.m.) across stroke subtypes and hinted at a secondary weekday pattern, with Monday showing a modestly higher incidence [27]. In a similarly designed study of community population, Wroe et al. (1992) [28], through the Oxfordshire Community Stroke Project, confirmed this diurnal pattern. They concluded that strokes are likely to happen in the morning hours, with a secondary peak rising in late night [28].

These independent findings were consolidated in Elliott’s (1998) [29] meta-analysis of over 11,000 stroke cases, which definitively established circadian rhythmicity in stroke onset. The pooled analysis demonstrated a marked clustering of events in the morning (approximately 6:00 a.m.–12:00 p.m.) and a nadir during the night (midnight–6:00 a.m.) [29]. Importantly, this pattern was consistent across ischemic and hemorrhagic strokes, as well as transient ischemic attacks, and persisted after controlling for confounders such as age and comorbid hypertension [29]. Elliott’s synthesis became the reference point for subsequent chrono-epidemiological investigations. In the 2000s, more attention started being paid to circadian patterns and the underlying causes of the onset risks. Many studies further refined the pattern by demonstrating dual surges in stroke onset, one in the morning and a smaller one in the evening, mirroring the bimodal daily variations in blood pressure and vascular tone [30,31,32]. These findings suggested that both behavioral (sleep–wake transitions, activity onset) and endogenous (autonomic and hormonal) factors converge to modulate cerebrovascular risk windows [4]. Collectively, over five decades of research have transformed the perception of stroke from a stochastic vascular event to one deeply influenced by biological time.

### 2.3. Circadian Patterns of Stroke Severity

**Figure 3:**
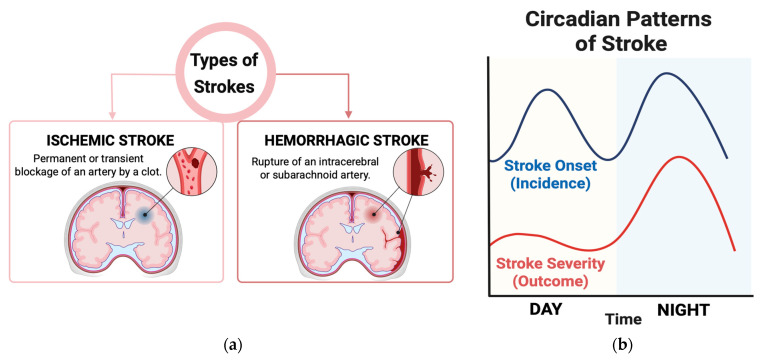
Stroke Subtypes and Circadian Patterns of Stroke. (**a**) Overview and schematic representation of the two major stroke subtypes. Ischemic stroke occurs when an artery supplying the brain becomes partially or completely occluded by a blood clot. Hemorrhagic stroke is the rupture of a cerebral vessel resulting in blood-borne factors leaking into the brain parenchyma; (**b**) Schematic illustrating the circadian pattern of stroke onset and severity. Epidemiological studies indicate stroke-onset rises with a morning peak and a smaller secondary peak in the night. In contrast, stroke severity has been reported to be greater during the late night or early morning hours. Created with Biorender.com.

Aside from stroke risk, a growing body of literature indicates that stroke severity and early outcome also exhibit a strong circadian pattern. While most early studies focused on incidence and timing, more recent work analyzed how the time of stroke onset is associated with neurological deficit, severity, early neurological deterioration (END), and other functional outcomes. One of the largest recent investigations analyzed over 17,000 patients with transient ischemic stroke, or TIA, and stratified the time of onset into night-onset stroke vs. day-onset stroke. The study revealed that night-onset strokes were prone to END and had a lower likelihood of favorable outcomes compared to day-onset strokes. The night-onset strokes also exhibited higher neurological severity with a significantly worse 3-month functional outcome [6]. Another clinical study pooled over 500 patients and found that infarct cores were larger at night than during the day, indicating a more severe and damaging stroke occurring at night (Figure 3).

Although the above clinical data came from human epidemiological studies, rodent studies display a similar pattern. In one study, the stroke was no different between strokes induced at ZT 0, 6, 12, or 18. However, they found the infarction sizes were distinct, with ZT 6 showing a larger infarct size and worse functional outcomes [33]. This was further supported by another preclinical study showing that the infarct size was significantly smaller in mice when stroke was induced at night (active phase) compared to day (inactive phase) [34]. Although many studies have made this circadian distinction clear, the reason underlying it remains unclear.

## 3. The Blood–Brain Interface in Stroke

A major determinant of stroke onset and severity may be the circadian state of the neurovascular system. The blood–brain interface (BBI) encompasses the collection of anatomical and molecular barriers that regulate communication between the central nervous system (CNS) and the systemic circulation. It is the brain’s first physical line of defense, and yet the first one to fall during a cerebrovascular event, such as a stroke [35]. To understand how this interface came to be recognized as a central regulator of cerebrovascular vulnerability, it is useful to first consider how the concept of the barrier itself originated and evolved.

### 3.1. Historical Evolution of the Blood–Brain Barrier

The concept of a physical and functional separation between the CNS and the peripheral circulation emerged more than a century ago through a series of seminal dye-permeability experiments. In 1885, Paul Ehrlich injected acidic dyes, such as trypan blue, into the systemic circulation of animals and noted that virtually all peripheral tissues became intensely stained while the brain and spinal cord remained conspicuously colorless. Ehrlich initially attributed this phenomenon to the low affinity of neural tissue for the dye rather than to the existence of a physiological barrier [36]. However, this observation ignited questions about selective exchange between the blood and brain.

A few decades later, in 1913, Edwin Goldman, a student of Ehrlich, conducted a complementary experiment: he infused trypan blue directly into the cerebrospinal fluid (CSF) and observed staining restricted to the brain and meninges but not in the peripheral organs. Goldman’s work provided the first experimental proof that the CNS was partitioned from the systemic milieu by a distinct barrier system [37,38]. Around the same time, Lewandowsky (1900) and Bouffard (1906) demonstrated that certain hydrophilic toxins or ions could not penetrate from blood to brain, reinforcing the notion of a specialized “Blut-Hirn-Schranke” (blood–brain barrier, BBB) [39,40].

Throughout much of the twentieth century, this barrier was conceived as a rigid anatomical wall, reflecting the reductionist view of the central nervous system as an immuno-privileged and isolated organ. Electron microscopy studies in the late 1900s identified tight junctions between cerebral endothelial cells, confirming a structural substrate for this impermeability. Consequently, the BBB was often depicted as a passive, static wall that merely prevented diffusion of plasma components into the neural parenchyma to prevent harm [41,42,43]. This “wall” model profoundly influenced both neuroscience and pharmacology, driving decades of research aimed at bypassing or transiently disrupting the barrier to enable or aid in drug delivery.

**Figure 4:**
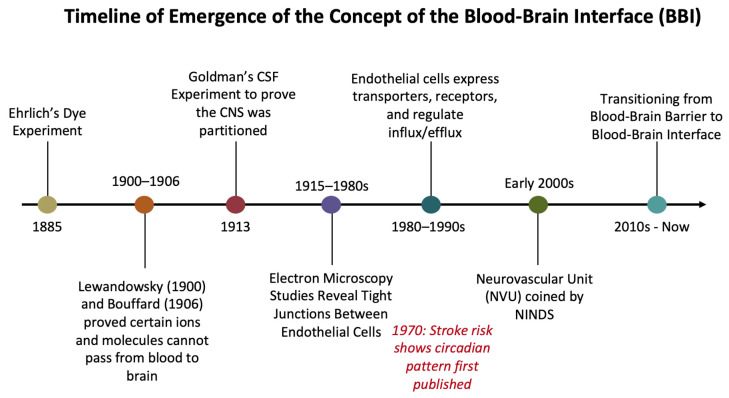
Timeline of the emergence of the concept of the blood–brain interface (BBI). Key experimental and conceptual milestones spanning from 1885 to the present are depicted chronologically. Ehrlich’s 1885 [36] dye exclusion experiment first demonstrated selective CNS impermeability, later confirmed by Lewandowsky (1900) and Bouffard (1906) [39,40], who showed that certain neurotoxic ions and large molecules cannot traverse from blood to brain. Goldman’s 1913 [38] cerebrospinal fluid injection studies established CNS compartmentalization. Electron microscopy studies (1915–1980s) revealed the structural basis of barrier function through tight junctions between neuroendothelial cells, while subsequent work (1980s–1990s) characterized the active transport properties of brain endothelial cells. Notably, the circadian patterning of stroke risk was first reported in 1970, foreshadowing the temporal dimension of BBI regulation. The neurovascular unit (NVU) framework was formalized by the National Institute of Neurological Diseases and Stroke (NINDS) in the early 2000s. Collectively, these advances drove a conceptual shift from the static “Blood–Brain Barrier” to the dynamic “blood–brain interface,” reflecting the bidirectional, regulated nature of CNS-periphery communication.

However, the subsequent advancements in molecular biology slowly transformed this perception. Beginning in the 1980s–1990s, it became evident that endothelial cells at the barrier express a wide range of transporters, receptors, and signaling molecules that actively regulate nutrient influx, waste efflux, and immune surveillance [44,45,46]. Astrocytic end-feet, pericytes, and the extracellular matrix were recognized as integral partners in maintaining and modulating barrier function [47]. Eventually, in 2001, a committee convened by the National Institute of Neurological Diseases and Stroke (NINDS) collectively termed this multicellular network the neurovascular unit (NVU) [48]. This broader perspective gave rise to the concept of the ‘blood–brain interface: a dynamic, multicellular communication hub, rather than a singular anatomical wall’ [49] (Figure 4).

### 3.2. Cellular and Structural Complexity of the NVU

**Figure 5:**
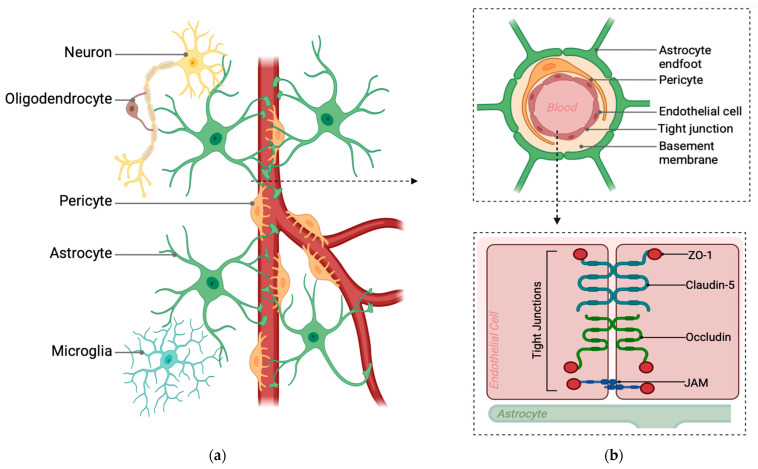
The Neurovascular Unit (NVU). (**a**) Schematic of a cerebral blood vessel illustrating key cellular components of the NVU. Pericytes are distributed along the abluminal surface of the endothelial vessel wall, while astrocytic end-feet extend from surrounding astrocytes to ensheathe the vessel. (**b**) Enlarged cross-sectional view of the NVU. Endothelial cells form the vascular lumen and are closely associated with pericytes that wrap around the vessel wall. Astrocytic end-feet project toward the vessel. The lower figure in (**b**) highlights the major tight-junction proteins located between two adjacent endothelial cells: zonula occludens-1 (ZO-1), claudin-5, occludin, and junctional adhesion molecules (JAM). Together, these form the junctions between the endothelial cells. Created with Biorender.com.

One of the first cracks in the “wall” paradigm was the recognition that the BBI is not a homogenous monolayer, but rather a multicellular and extracellular composite structure [47,48,50,51]. Physiologically, the BBI consists of the NVU. At the core of the BBI lies the brain microvascular endothelium [52], characterized by continuous tight junctions and an exceptionally low rate of transcytosis. Brain blood vessel endothelial cells differ in morphology and function from the peripheral endothelial cells. They express a specialized repertoire of tight-junction proteins, including claudin-5, occludin, and zonula occludens-1 (ZO-1). These junctions separate the blood (luminal) and the brain (abluminal), restricting paracellular flux [8] (Figure 5). Additionally, BBI endothelial cells have a higher amount of mitochondria compared to peripheral endothelial cells, indicating a need for high energy demand, likely for transport [53]. The endothelial cells sit atop a basement membrane enriched in laminins, collagen, and nidogen. This membrane provides structural support and signaling cues that shape endothelial phenotype and vascular permeability [54].

Ensheathed on the endothelial cells are pericytes, a type of mural cell that wraps around the capillary to regulate blood flow. Similar to a smooth muscle cell, pericytes can contract and relax, allowing vasoconstriction and vasodilation [55,56]. Pericytes have also been shown to communicate with endothelial cells, contributing to the BBI permeability. During vessel formation, endothelial cells secrete platelet-derived growth factor-B (PDGF-B), which binds to PDGF-R on pericytes for recruitment. Loss of PDGF-B or PDGF-R has been shown to result in pericyte deficiency, leaky microvessels, and disrupted endothelial morphology [55,57,58]. As pericytes establish contact, reciprocal communication maintains vessel homeostasis. Transforming growth-factor-βeta (TGF-β), produced by both cell types, signals through the ALK1/ALK5-Smad pathways to regulate angiogenesis and pericyte contractility. Additionally, angiopoietin-1 (Ang-1) and angiopoietin-2 (Ang-2) are released, mediating inflammatory responses. Ang-1, primarily released by pericytes, binds to Tie2 receptors on the endothelial cells, enhancing tight junction formation, suppressing inflammatory permeability cues, and maintaining barrier integrity [59,60]. On the other hand, Ang-2 stimulates this process during stress or injury [55]. Contact-dependent mechanisms also contribute to pericyte-endothelial communication. Direct gap junctions composed of connexin-43 allow bidirectional exchange of ions and small metabolites, synchronizing calcium and metabolic signaling between the two cell types [61,62].

Projecting from the brain parenchyma, astrocytes are the multifunctional star-like cells of the brain. Astrocytes form a perivascular sheath with their end-feet processes around the microvessels [8]. These end-feet are enriched in aquaporin-4 (AQP4) and Kir4.1 potassium channels, which coordinate water and ion flux across the neurovascular unit (NVU). This anatomical arrangement contributes to regulating vascular tone, osmotic balance, and permeability [63,64]. The astrocytes also serve as a signaling interface between neurons and endothelial cells. At the molecular level, the astrocytes release a variety of paracrine factors, including basic fibroblast growth factor (bFGF), Ang-1, TGF-β, and vascular endothelial growth factor (VEGF). These factors modulate endothelial tight-junction formation and transporter expression [65,66]. Thus, astrocytes function as bidirectional modulators of BBI permeability, capable of reinforcing or dismantling the barrier in response to local environmental cues.

### 3.3. The NVU in Stroke

Stroke is an acute cerebrovascular event defined by the sudden disruption of cerebral blood flow due to vessel occlusion (ischemic stroke) or rupture (hemorrhagic stroke), resulting in rapid neuronal dysfunction and tissue injury (Figure 3) [67]. Within seconds of a stroke, the brain microvascular endothelium experiences profound metabolic and inflammatory stress. In ischemic stroke, a cerebral artery becomes blocked, causing blood flow to drop sharply. This abrupt obstruction initiates a cascade of molecular, vascular, and immune events that unfold over seconds to days, determining the degree of neurological impairment [68,69]. The BBI is one of the first structures affected and plays a defining role in the progression of injury [10,70].

Immediately after artery occlusion, oxygen and glucose delivery stop, causing neurons, astrocytes, oligodendrocytes, and endothelial cells to undergo rapid energy failure [71]. ATP depletion inactivates ion pumps, causes membrane depolarization, and triggers necrotic or programmed cell death. It has been reported that within seconds, millions of neurons rapidly die, contributing to the fatality and long-term disability associated with stroke [72,73]. These dying cells release a burst of damage-associated molecular patterns (DAMPs), which include extracellular ATP, mitochondrial DNA, HMGB1, reactive oxygen species (ROS), and calprotectins. These DAMPs act as danger signals and engage with pattern-recognition receptors (PRRs) expressed on microglia and perivascular macrophages [74,75]. Microglia throughout the parenchyma and around vessels detect DAMPs via toll-like receptors, RAGE, and NOD-like receptors [76]. Endothelial cells also express similar PRRs and respond to high extracellular ATP via P2X7 [77]. This triggers intracellular signaling cascades, such as NF-kB translocating to the nucleus, MAPKs becoming activated, and early STAT signaling. Even before the peripheral immune system reacts to signals in the circulation, the microglia, endothelial cells, and astrocytes have moved toward a more pro-inflammatory state, upregulating transcription of pro-inflammatory cytokines (IL-6, TNF-α, IL-1β) and chemokines (CCL2, CXCL1) [78,79]. In this immediate phase, much of the inflammatory activity is coming from the central nervous system. Microglia begin to retract their processes, and endothelial cells start to alter phosphorylation, changing tight-junction protein expression [76,80,81].

As endothelial cells initiate the peripheral immune response, they increase expression of adhesion molecules [82,83]. The early inflammatory process intensifies, and the cytokines released by the astrocytes and microglia, especially TNF-α, IL-1β, and IL-6, act directly on the endothelium to induce high expression of P-selectin and E-selectin [84,85,86]. These carbohydrate ligands act as molecular “hooks” to capture circulating leukocytes. Almost simultaneously, the endothelium upregulates ICAM-1 (intracellular adhesion molecule 1) and VCAM-1 (vascular cell adhesion molecule-1), which mediate firm adhesion through binding to leucocyte integrins (LFA-1) [86,87]. These adhesion molecules do not simply appear; their expression is coordinated with chemokine production. Endothelial cells also secrete chemokines, forming a gradient that stimulates immune cell trafficking [84,88]. Luminal chemokine gradients activate leukocyte G-protein coupled receptors (GPCRs), triggering the inside-out signaling cascade required for leukocyte integrins to convert from low-affinity to high-affinity conformations, allowing them to bind to adhesion molecules [89,90]. Additionally, neuroendothelial cells, already under profound metabolic and oxidative stress due to hypoxia, undergo signaling and cytoskeletal changes that alter tight-junction organization. Activation of kinase pathways and cytoskeletal contraction leads to phosphorylation and redistribution of junctional proteins, facilitating leukocyte transmigration. This marks the transition of the vascular sealed barrier into a regulated entry point, defining early stroke inflammation [91,92].

Neutrophils respond to this molecular entry by quickly binding to the adhesion molecules. As neutrophils roll and adhere, they transmigrate through the vessel carrying a destructive enzymatic arsenal. Matrix metalloproteinase-9 (MMP-9) and ROS are released by neutrophils, degrading the basement membrane of microvessels and leading to further breakdown [93,94]. This increases the risk of hemorrhagic transformation, a hallmark of increased stroke severity [93,94,95]. Once inside the parenchyma, the neutrophils begin forming neutrophil extracellular traps (NETs), web-like structures of decondensed chromatin, histones, and proteases. These NETs trap platelets and erythrocytes, promoting microvascular thrombosis. This further compromises BBI integrity and expands tissue damage [96,97].

After the first neutrophil invasion, monocytes and macrophages start to dominate the immune cell invasion of the stroke brain, marking the subacute secondary inflammatory process. Depending on the severity of the stroke, these cells can either increase vascular dysfunction and amplify immune activation or initiate recovery pathways [98]. This recruitment is primarily governed by the release of chemokines from the reactive astrocytes, activated microglia, and inflammatory neuroendothelial cells. This draws inflammatory monocytes from the bloodstream to the injured brain, and they interact with the neuro endothelium to transmigrate through tight junctions. Upon entering the parenchyma, the monocytes transition into monocyte-derived macrophages, joining the resident microglia in the inflammatory process and rapidly releasing cytokines [99,100,101].

At the tissue level, macrophages and microglia start engaging in phagocytosis, marking the beginning of the recovery process [98]. However, phagocytosis is often inefficient during the early inflammatory window, as the high levels of pro-inflammatory cytokines and reactive oxygen species (ROS) prolong the DAMPs within the affected area [102]. At this stage, T cells begin infiltrating the ischemic brain. Specifically, CD4+ Th1 cells secrete IFN-γ, amplifying macrophage activation and increasing leukocyte recruitment [102,103,104,105,106]. Th17 cells release IL-17A, a cytokine that synergizes with TNF-α and IL-1β to promote tight-junction protein degradation and increase paracellular permeability [103,105]. These reactions together mark the secondary injury, contributing to stroke severity and damage.

Over several days, the macrophages then slowly start to shift toward a reparative state, a transition essential to resolution and tissue repair. As the necrotic tissue starts to clear, DAMPs diminish within the parenchyma [107]. As a result, macrophages and microglia start to release IL-10 and TGF-β, anti-inflammatory mediators, reducing the immune activation [102]. Anti-inflammatory cytokines start to suppress the NF-kB signaling and downregulate pro-inflammatory molecules [108,109]. Growth factors, such as VEGF, IGF-1, and BDNF, increase, promoting angiogenesis and vascular stabilization. These factors contribute to the restoration of the endothelial tight-junction proteins, suppressing MMP activity, and rebuilding the BBI [110]. However, when this resolution is impaired, such as during aging or circadian-vulnerable windows, BBI instability can be perpetuated and worsen long-term outcomes.

## 4. Circadian Regulation of the BBI

Circadian rhythms govern a multitude of physiological processes and have been implicated in the regulation of the BBI. A growing body of evidence indicates that the BBI is a dynamically rhythmic system whose molecular and functional properties oscillate across a 24 h day. The canonical transcription-translation feedback loops are present in endothelial cells, astrocytes, and pericytes [111]. Recent research supports a key role for circadian modulation of susceptibility of the BBI to stroke severity and immunomodulation with time of day.

### 4.1. The Molecular Circadian Clock

A landmark study by Zhang et al. demonstrated that the activity of the efflux transporter, P-glycoprotein (ABCB1/MDR1), at the BBI oscillates in mice, with peak activity occurring during the active phase (night or lights-off) [112]. The authors showed that this rhythm depends on the neuroendothelial molecular clock: in mice whose Clock gene has been deleted, the efflux rhythm is lost. Mechanistically, they linked BMAL1-mediated transcriptional regulation of TRPM7, a Mg^+2^ transporter, to rhythmic intracellular Mg^+2^ levels, which in turn influence ABCB1-mediated efflux capacity [112]. This is notable because the transcript levels of ABC transporters themselves did not show strong rhythmicity; rather, it was the intracellular Mg^+2^ that was robustly rhythmically modulated [112].

Some work has been performed to evaluate circadian changes in tight-junction proteins in the periphery, but not in the brain. One study found that mRNA expression of two tight-junction proteins, occludin and claudin-5, oscillates according to time of day in the mouse intestine. However, in Per2 mutant mice, the tight-junction protein mRNAs lose their oscillations, increasing overall intestinal permeability [113]. Circadian disruption due to imposed irregular environmental lighting cycles causes increased permeability in the intestinal epithelial barrier in mice. In control conditions, occludin mRNA expression exhibits circadian oscillations, but is disrupted in ClockΔ19/Δ19 mice [114]. Some work on understanding molecular uptake indicates TNF-α, leptin, β-amyloid, and prostaglandin D2 are rhythmically active based on transport changes in the BBI [111,115,116,117].

There has not been a study conducted on whether the permeability of the BBI oscillates according to time of day [113,114]. Additionally, many of these studies have been conducted in rodent models. Reconceptualizing the BBI as dynamic has profound functional and translational ramifications. Because barrier tightness and transporter activity oscillate, there may be periods during the circadian cycle when the interface is more permissive to circulating solutes or a more robust barrier, creating vulnerability windows or windows protected from stroke.

### 4.2. Astrocytes and Microglia Are Under Circadian Control at the BBI

Beyond the whole barrier, many studies have found that astrocytic and microglial responses are under circadian control. Astrocytes have one of the most robust clocks within the CNS [47,84,85]. A key interface-linked astrocyte regulator, aquaporin 4 (AQP4), localization and glymphatic flow exhibit strong circadian regulation. AQP4 typically has a polarized localization at the tips of processes that engage the neuroendothelium. This polarization is also the highest during the rest phase. Genetically deleting AQP4 eliminates this difference [118]. At the cortical level, astrocytic intracellular Ca^2+^ exhibits distinct patterns, being reduced during natural sleep [119,120]. Additionally, the daily release of ATP, an important aspect of astrocytic immune function, is rhythmic [121].

Microglia, the brain’s resident innate immune cells, are also under circadian control. Microglia exhibit rhythmic inflammatory cytokine release, including TNF-α, IL-1β, and IL-6, with peak expression occurring during the rodent rest phase [122,123]. Disruption of core clock genes through siRNA-mediated knockdown or in circadian mutant mice abolishes these rhythmic cytokine patterns [124]. In addition to cytokine secretion, microglial phagocytic activity displays a diurnal pattern, with increased phagocytosis during the dark (active) phase in the hippocampus [125]. Microglia-specific knockdown of *Bmal1* increases synaptic engulfment in the mouse hippocampus, and *Bmal1* knockdown in vitro via siRNA similarly enhances phagocytic activity [126,127]. Microglial morphology changes rhythmically, as well. The influx and clearance of glymphatic cerebral spinal fluid (CSF) exhibit endogenous circadian rhythms, peaking during the rest phase [118,128]. Branching complexity is higher during sleep and reduced during wakefulness, suggesting time-of-day-dependent shifts in surveillance versus reactive states [129].

### 4.3. The Peripheral Immune System Is Under Circadian Control

Outside of the CNS, most of the major immune cell populations exhibit circadian rhythms. In mice, leukocyte adhesion and migration in the bone marrow and skeletal muscle microvasculature peak during the active phase at night. These time-of-day-dependent changes are driven by rhythmic sympathetic adrenergic innervation of the vasculature, which regulates endothelial adhesion-molecule expression and leukocyte trafficking [130]. Pro-migratory molecules on endothelial cells and leukocytes exhibit tissue-specific rhythmic expression that determines time-of-day-dependent homing [131,132]. These rhythms are tightly linked to adhesion molecules and chemokines: endothelial ICAM-1, VCAM-1, selectins, and chemokines show distinct circadian oscillations [131,132]. Monocytes and macrophages are clock-sensitive at multiple levels. Around eight percent of the macrophage transcriptome is circadian-controlled [133]. Further, macrophage cytokine expression and release are also circadian-regulated with distinct oscillations when challenged with an inflammatory agent [133]. Additionally, the macrophage NLRP3 inflammasome is under circadian regulation, in part through clock-dependent control of mitochondrial metabolism and ROS production. Together, they influence inflammasome activation [134].

Altogether, these findings mean that when a stroke occurs, the peripheral immune system is in a specific phase-state, with differing levels of circulating inflammatory activation. Future research correlating immune activation, BBI permeability, and transport is needed to understand the intricacies of the circadian regulation of stroke onset and severity. They directly impact how rapidly and vigorously immune cells are recruited to the injured BBI.

## 5. Aging and BBI Dysfunction in Stroke

Age is the single strongest non-modifiable risk for stroke. The aging BBI undergoes progressive structural weakening, impaired BBI homeostasis, altered immune signaling, and diminished capacity to resolve inflammation. Collectively, these age-related changes convert the BBI from a tightly regulated barrier into a vulnerable fence that amplifies injury.

### 5.1. Aging Impacts Stroke-Induced Inflammation

At the systemic level, aging is accompanied by “inflammaging,” a term coined for chronic low-grade elevation of pro-inflammatory mediators, such as IL-6, TNF-α, and IL-1B. This is a product of the NF-kB pathway and NLRP3 activation within vascular cells, with profound implications [135,136]. A change in the BBI microenvironment into a state of chronic inflammation alters how the immune system engages in disease. In an experimental ischemic stroke model, aged mice subjected to cerebral middle artery occlusion (MCAO) had a higher mortality rate compared to young mice. However, aged mice showed a relative decrease in infarct size compared to young mice, but had a higher hemorrhagic transformation. This suggests that although the infarct size was smaller, the induced stroke was severe [11]. Additionally, a significantly higher percentage of circulating neutrophils was found in the blood of aged mice after stroke, which indicates an age-related exacerbation of the bone marrow response. Leukocyte infiltration and neutrophil migration were significantly increased in aged mice compared to young, highlighting an altered immune cell response [11]. Aging fundamentally disrupts the molecular composition and membrane localization of tight-junction proteins. When aged mice underwent MCAO, they exhibited higher infarct sizes, increased BBI leakage, and increased pro-inflammatory cytokine expression in the ischemic brain. Interestingly, both ZO-1 and claudin-5 had decreased protein expression in the brain of aged mice experiencing stroke compared to young mice, in parallel to BBI leakage [137].

### 5.2. Aging-Induced Vulnerability of the BBI

**Figure 6:**
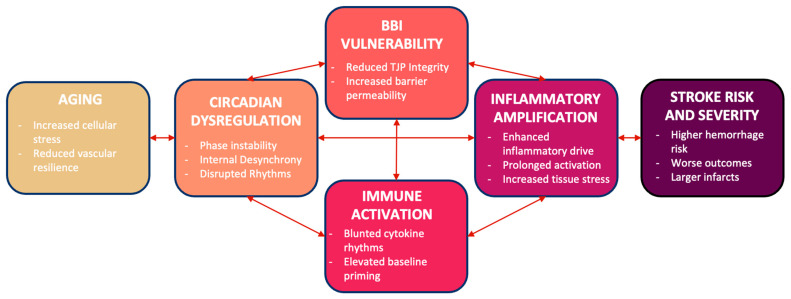
Conceptual Framework. Aging contributes to increased cellular stress and reduced vascular resilience, which contributes to circadian dysregulation. Disruption of circadian rhythms drives altered immune activation, leading to chronic low-grade inflammation. These processes converge to increase stroke susceptibility and risk.

One of the central reasons stroke outcomes worsen with age is that the BBI becomes structurally and molecularly compromised, long before a cerebrovascular event occurs (Figure 6). Cerebral microvessels in aged brains exhibit reduced capillary density, impaired endothelial tight junctions, and increased oxidative stress. Nitric oxide (NO) bioavailability is crucial for endothelial cell function and regulation. NO is synthesized by endothelial nitric oxide synthase (eNOS), which decreases with age [138]. Reduced eNOS activity leads to decreased NO production, down-regulating caveolin-1 (CAV-1), a negative regulator of eNOS. This negative feedback loop further promotes endothelial dysfunction, increased leukocyte adhesion, and loss of vascular reactivity. CAV-1 accumulation also enhances transcellular trafficking, contributing to a leakier phenotype in aged microvessels. Neuroendothelial tight-junction proteins decline with age, and their membrane localization becomes more disorganized, which weakens paracellular sealing. Aging increases endogenous IgG extravasation and BBI permeability [137,139]. Neuroendothelial cells also lose key physical support during aging, with pericytes becoming more shriveled. Pericytes gradually diminish in number, which destabilizes neuroendothelial junctions and impairs neurovascular coupling [140,141,142].

### 5.3. Circadian Senescence: Aging and the Decline of Molecular Rhythmicity

These studies usually do not take circadian time into account. Aging profoundly affects circadian rhythmicity across the brain and periphery, leading to a global decline in molecular clock amplitude, phase precision, and oscillator coupling. One of the earliest hallmarks of circadian aging is the deterioration of the central circadian clock tissue in the brain’s suprachiasmatic nucleus (SCN). Aged SCN neurons show reduced electrical synchrony, weakened rhythmic firing, and dampened oscillations [143]. This reflects an intrinsic loss of cellular rhythmicity. Aging also impairs neurotransmitter signaling within the SCN, including vasoactive intestinal peptidergic (VIP) and GABAergic communication. This weakens intracellular coupling and reduces the robustness of daily rhythms [143,144,145,146]. At the molecular level, core clock-gene expression displays reduced amplitude. BMAL1, especially, declines significantly with aging. BMAL1-deficient mice show accelerated system-wide aging phenotypes, including BBI dysfunction, altered rhythms, and loss of functional NVUs [111,112,147,148].

Critically, this age-related decline in BMAL1 is not a passive process but is actively driven by the inflammatory environment of aging. The chronic low-grade inflammation characteristic of inflammaging engages NF-κB signaling, whose canonical subunit, RELA, directly represses BMAL1/CLOCK transcriptional activity at E-box elements. This results in RELA competing with CLOCK for BMAL1 binding, dampening circadian amplitude [149]. In vascular endothelial cells specifically, NF-κB-driven promoter methylation of BMAL1 suppresses its transcription; the resulting BMAL1 loss has been associated with enhanced p65 phosphorylation through CLOCK-dependent mechanisms, further amplifying NF-κB inflammatory output [150]. This establishes a self-reinforcing vicious cycle between inflammaging and circadian erosion directly at the endothelium. Consistent with this, BMAL1 expression declines specifically in endothelial cells during aging, with corresponding loss of circadian oscillations in downstream vascular targets [151]. Aging does not just weaken the interface structurally, but actively desynchronizes it through an inflammatory feedback loop.

## 6. Integrative Framework and Conclusions

A crucial methodological challenge in this field lies in aligning the biological phases of humans and rodents. Humans are diurnal, while rodents are nocturnal; thus, a stroke occurring during the early morning in humans, the onset of the active phase corresponds to the early night-phase in rodents, which represents their rest period (Figure 1). By standardizing reporting and analysis of the temporal phase, the field can move toward harmonized datasets capable of mapping equivalent biological times between species [34]. Most preclinical stroke models are assessed during the day, the inactive phase of rodents, but the active phase for humans. This introduces a systematic bias that likely amplifies infarct size relative to injuries induced during the dark, active phase [34]. Reporting the Zeitgeber Time (ZT) of every experimental procedure, including injury induction, imaging, and tissue collection, along with documentation of the light–dark cycle, feeding schedule, and entrainment conditions, is fundamental for reproducibility. Similarly, clinical studies must include the precise time of stroke onset, blood sampling, imaging, and therapeutic intervention. This information would enable meta-analyses that correlate outcomes with internal circadian phase rather than arbitrary wall-clock time, thereby identifying the true temporal windows of vulnerability.

Time-of-day-dependent approaches to stroke prevention and treatment are already emerging. Chronotherapy, the administration of interventions or drugs timed according to circadian biology, is gaining traction as a clinically relevant strategy [12,13]. Circadian rhythm disruption can aggravate brain injury severity, and conversely, stroke itself can alter core clock gene expression in the ischemic brain [152,153]. This bidirectional interaction between cerebrovascular injury and the molecular clock warrants further investigation, as clock genes themselves may represent viable therapeutic targets.

One of the most established stroke therapies, intravenous thrombolysis, may be particularly sensitive to circadian timing. The fibrinolytic system follows a robust circadian pattern: tissue plasminogen activator (tPA) and its inhibitor PAI-1 exhibit opposing diurnal oscillations, with PAI-1 peaking in the early morning and tPA activity at its nadir, temporally coinciding with the morning peak of ischemic stroke incidence [154,155,156]. Vilas et al. demonstrated that patients receiving rt-PA during daytime hours had significantly higher rates of cerebral artery recanalization and more favorable functional outcomes compared to those treated at night [157]. Consistent with this, rodent studies suggest that treatment administered during the inactive phase significantly reduces infarct volume compared to intervention during the active phase [158]. At the molecular level, melatonin and its receptor agonists, acting via MT1/MT2 signaling, reduce infarct volume and improve functional outcomes through antioxidant, anti-inflammatory, and anti-apoptotic mechanisms in preclinical stroke models [159,160,161]. REV-ERBα agonists such as SR9009 confer phase-dependent neuroprotection through suppression of NLRP3-mediated neuroinflammation and Nrf2 pathway activation [158]. Together, these converging findings argue that circadian biology should be explicitly integrated into stroke therapeutic design.

Capturing the temporal behavior of the BBI requires multimodal, phase-resolved, and physiologically relevant experimentation. In vitro platforms, including primary and induced pluripotent stem cell (iPSC)-derived endothelial–pericyte–astrocyte co-cultures, enable continuous monitoring of molecular interactions between cell types. A significant model is the “BBI on chip” that reproduces in vivo conditions in vitro [162,163]. Recent advancements in BB-on-chip technology have integrated iPSC-derived cellular components, physiological shear stress, and 3D matrices to reconstitute and better mimic the human NVU in vitro [162,164,165]. Microfluidic platforms incorporating the NVU have demonstrated their utility in validating therapeutic interventions targeting CNS diseases [166,167]. For circadian BBI research specifically, microfluidic platforms hold promise because their capacity for long-duration, continuous monitoring is uniquely compatible with the timescales of circadian oscillations. This provides real-time readouts of the interface integrity that a 2D system cannot provide. Rodent studies utilize fluorescent dyes and tracers to quantify changes in BBI uptake. Despite recent advances, several knowledge gaps persist. However, direct evidence for human BBI rhythmicity remains very limited. Therefore, it is critical to apply different approaches to study the human BBI. Moreover, prevalent comorbidities, such as hypertension, diabetes, and sleep disorders, disrupt vascular clock function and are likely to synergize with aging to exacerbate temporal vulnerability.

In conclusion, we have presented a summary of circadian modulation of stroke onset and severity, a simplified version of the molecular mechanisms of the neuroinflammatory cascade within the BBI after an ischemic stroke, the circadian regulation of the BBI, and how age may contribute to the likelihood of severe strokes. Importantly, aging functionally and mechanistically impairs the BBI by dampening the clock and driving chronic inflammation. As the clock desynchronizes over the lifetime, inflammatory tone rises; it destabilizes the interface, which progressively widens the windows of vulnerability. This feed-forward cycle offers a mechanistic explanation for the disproportionately higher stroke risk and severity seen in the aging population. We have noted windows of vulnerability that the circadian cycle creates, which may enable us to understand why strokes are more likely to occur at specific times of the day and why time-dependent strokes may favor worse outcomes. A deeper understanding of the relationship between the circadian clock at both the cellular and systems level will elucidate the nature of BBI weaknesses in temporal vulnerabilities to stroke and enable the development of a new class of effective treatments.

## Data Availability

No new data were created or analyzed in this study. Data sharing is not applicable to this article.

## Abbreviations

The following abbreviations are used in this manuscript:

ANG-1	Angiopoietin-1
AQP4	Aquaporin-4
BBI	Blood–Brain Interface
BBB	Blood–Brain Barrier
BFGF	Basic Fibroblast Growth Factor
BMAL1	Brain and Muscle ARNT-Like 1, a core clock gene
CCGs	Clock-Controlled Genes
CLOCK	Clock Circadian Regulator, a core clock gene
CNS	Central Nervous System
CRY	Cryptochrome
END	Early Neurological Deterioration
eNOS	Endothelial Nitric Oxide Synthase
ICAM-1	Intercellular Adhesion Molecule 1
MMP-9	Matrix Metalloproteinase-9
NETs	Neutrophil Extracellular Traps
NVU	Neurovascular Unit
PDGFR	Platelet-Derived Growth Factor Receptor
PER	Period, a core clock gene
ROS	Reactive Oxygen Species
SCN	Suprachiasmatic Nucleus
TGF-β	Transforming Growth Factor-Beta
TTFL	Transcription-Translation Feedback Loop
VIP	Vasoactive Intestinal Peptide
ZO-1	Zonula Occludens-1
